# Primary care physicians’ perceptions of hypertension management in Qatar: A qualitative study using the theoretical domains framework

**DOI:** 10.1371/journal.pone.0318527

**Published:** 2025-04-24

**Authors:** Nada Abdelkader, Ahmed Awaisu, Hazem Elewa, Samya Ahmad Al Abdulla, Maguy Saffouh El Hajj

**Affiliations:** 1 Clinical Pharmacy and Practice Department, College of Pharmacy, QU Health, Qatar University, Doha, Qatar; 2 Professor and Department Head, Clinical Pharmacy and Practice Department, College of Pharmacy, QU Health, Qatar University, Doha, Qatar; 3 Associate Professor, Doctor of Pharmacy degree director, College of Pharmacy, QU Health, Qatar University, Doha, Qatar; 4 Senior Consultant Family Physician, Executive Director of Operations, Primary Health Care Corporation, Doha, Qatar; 5 Clinical Associate Professor, Clinical Pharmacy and Practice Department, College of Pharmacy, QU Health, Qatar University, Doha, Qatar; Taylor's University - Lakeside Campus: Taylor's University, MALAYSIA

## Abstract

Antihypertensive medications are known to reduce the incidence of hypertension complications. While the benefits of these medications are recognized, the factors influencing the prescribing practices of primary care physicians in Qatar have not been assessed. This study investigated Qatar primary care physicians’ experiences, practices, and perceptions regarding hypertension management. The study further evaluated the factors that influence their prescribing behaviors and identified strategies for improving hypertension management in primary healthcare settings. A qualitative case study was conducted through one-to-one semi-structured interviews with physicians practicing at the 31 Primary Health Care Corporation (PHCC) centers in Qatar. Due to the small population size, there was no need for sample size calculation and whole population sampling was used (N=179). Physician leads at each PHCC center helped identify eligible participants. Study information were shared via email and interested candidates were contacted to arrange for interviews at their convenience. The interview guide questions were designed based on the 14 domains of the Theoretical Domains Framework (TDF) of behavior change. Interviews were conducted virtually and transcribed verbatim. Thematic analysis was undertaken using inductive and deductive approaches. Twelve themes emerged, including physicians’ knowledge, skills, goals and role in hypertension management, confidence about prescribing decisions, optimism that prescriptions yield positive results, methods to ensure the appropriateness of prescriptions, emotions, and experiences when prescribing antihypertensives, perceived barriers, and facilitators when prescribing and social influences. Strategies for improving hypertension management were also suggested. In conclusion, multiple factors were identified that affect participants’ antihypertensive prescribing. Further research is needed to evaluate the suggested strategies’ effectiveness and to explore other improvements.

## Background

Around 1.28 billion persons worldwide were diagnosed with hypertension in 2023 according to the World Health Organization (WHO) [1]. Based on a 2015 survey, a global report on non-communicable diseases (NCDs) indicated that on average one in four men and one in five women experienced high blood pressure [2]. If not detected early and managed effectively, hypertension is one of the leading preventable causes for morbidity and death [3, 4].

A number of national and international clinical practice guidelines were developed to offer guidance for the treatment of hypertension [5–8]. The National Institute for Health and Care Excellence Hypertension in Adults Guideline (NICE), the American College of Cardiology/American Heart Association (ACC/AHA), the European Society of Cardiology/European Society of Hypertension Guideline (ESC/ESH), and the Eighth Joint National Committee Guideline (JNC8) are a few examples [5–8]. In addition to guidelines’ recommendations, several factors may affect physicians’ prescribing practices for hypertension management. These include their previous experiences, knowledge, and skills, patients’ socioeconomic status, medication adherence, medication profile and tolerability, as well as medication advertising, pricing, cost-effectiveness, and availability. In addition, the social and cultural context of the healthcare system and organizational factors play vital roles [9, 10].

A review of studies conducted in the State of Qatar and published between 1982 and 2019 found that 32% of people had hypertension [11]. According to estimates, 57% of deaths in Qatar in 2019 were attributed to cardiovascular diseases and high systolic blood pressure [12]. Qatar’s Ministry of Public Health (MOPH) has aligned its national strategy with the global target set by the WHO which is to reduce uncontrolled high blood pressure by 25% by 2025 [13]. In Qatar, the majority of hypertension patients are treated in government clinics run by the Primary Healthcare Corporation (PHCC) [14]. The JNC8 and NICE guidelines’ recommendations serve as the foundation for the PHCC’s hypertension management guidelines [14, 15]. However, there is a general lack of information regarding the practices and perspectives of primary care physicians in Qatar concerning hypertension management and the factors influencing their choice of antihypertensive medications. Therefore, the study aimed to: [1] investigate Qatar primary care physicians’ experiences, practices, and perceptions related to hypertension management, [2] evaluate the factors influencing their prescribing behaviors in this regard, and [3] potentially identify/propose strategies to improve hypertension management in primary care settings.

## Methods

This study was reported as per the Consolidated Criteria for Reporting Qualitative Research (COREQ) [16].

### Study setting

The study was conducted at primary health centers operated by PHCC in Qatar. This encompasses 31 centers throughout Qatar, covering the Central, Western, Southern and Northern regions [17]. The primary health centers in Qatar are responsible for managing the majority of outpatients suffering from such as hypertension in the country.

### Study design

A qualitative case study was conducted using one-to-one semi-structured virtual interviews to explore physicians’ experiences, practices, and perceptions. Semi-structured interviews were chosen over focus groups (FGs) due to the varying schedules and availability of physicians at the health centers. The qualitative case study is the most common and deemed the most suitable method when exploring behavioral phenomena and perceptions [18]. The interview guide and the data analysis were designed based on a well-established theoretical framework, the Theoretical Domains Framework (TDF) [19].

### Study population and participants’ recruitment

All primary care physicians from the 31 PHCC centers who were involved in hypertension management were eligible to participate. Physicians not engaged in managing hypertensive patients were excluded. There were 179 eligible physicians at the time of the study. Due to the small population size, there was no need for sample size calculation and whole population sampling was used. Physician leads at each PHCC center were approached to help identify eligible participants for the study. Emails including information about the study and its objectives were sent to potential participants. Interested candidates were subsequently contacted via email or phone to arrange for interviews at their convenience.

A day before the scheduled interviews, physicians were contacted again to confirm their attendance. Data collection continued until data saturation was achieved [20].

### Interview guide

The interview guide was structured based on the TDF, which was developed through consensus and validation by an expert panel to integrate multiple behavior change theories [19]. The TDF comprises 14 domains with 84 determinants grounded in psychological theory and has been widely utilized in healthcare to understand behaviors for designing complex interventions [21–24]. Moreover, the framework is derived from 33 psychological theories and 128 theoretical constructs developed into 14 overarching domains of behavioral determinants [25], offering a robust theoretical basis for implementation studies [21]. Therefore, the TDF was deemed a suitable framework to assess the physicians’ behavior and explore their perspectives on prescribing antihypertensives and managing hypertension. The behavior of interest for the current study was the prescribing practices of antihypertensive medications in PHCC centers in Qatar. Each question in the interview guide was carefully designed to be in line with each of the 14 TFD domains.

### Validation of the interview guide

The interview guide was reviewed by a panel of experts consisting of two faculty members from the College of Pharmacy, Qatar University, with experience in qualitative research. The main purpose of this validation process was to ensure that the questions were appropriate and comprehensive. Subsequently, the interview guide was revised based on their feedback and suggestions.

### Data collection

The semi-structured interviews were conducted between December 2021 to May 2022. Given that English is one of the primary languages of communication in Qatar, the interviews were conducted in English. Using the validated interview guide, one researcher (NA), who is a female Master of Pharmacy candidate and trained by the study's principal investigator in interview facilitation (MH), moderated all sessions of the interviews. MH took notes during the interviews.

The research team included MH: female faculty member and project leader, PharmD degree holder with experience in pharmacy practice research, AA and HE: male faculty members, and PhD degree holders. The researchers did not have any established relationships with the participants and had extensive experience in qualitative research.

Microsoft Teams™ software was utilized for virtual interviews, which were recorded and subsequently transcribed for analysis. The interview link was emailed to the participants who consented to participate at least three days prior to the interview. Before the interview, informed consent forms were sent to participants, who signed and scanned the forms and returned them to the research team. Data collection continued until saturation was achieved.

### Transcribing of interviews

The interviews were audio-recorded and transcribed using Microsoft Teams™ software. The transcribed documents were carefully reviewed and compared with the original audio recordings and field notes to ensure accuracy by the lead principal investigator (MH) and the study researcher (NA). Then they were shared with participants for review.

### Qualitative data analysis

Thematic analysis was conducted using both deductive and inductive methods manually Initially, a deductive approach was employed using the TDF, with its 14 domains to identify relevant themes [19]. Additionally, the transcripts underwent inductive analysis using the following six steps: familiarizing with the data; creating preliminary codes; looking for related themes; reviewing themes; defining themes; and writing the results [26]. The lead principal investigator (MH) and study researcher (NA) independently analyzed the data manually. Through an iterative process, the research team reviewed the findings, discussed and compared codes and themes, challenged individual assumptions, and refined the analyses as needed, until consensus was reached.

### Quality measures

To ensure the trustworthiness of the research findings, four pillars of quality measures were used: credibility, transferability, dependability, and confirmability [27]. Lincoln and Guba define dependability as the stability of findings over time. While confirmability refers to the extent to which other researchers are able to confirm the study results and that the results are not based on the researcher’s bias rather than actual data [27]. Dependability and confirmability were addressed by having an audit trail, and a detailed description of the study methodology, and by building a database that contains all the records obtained as part of the project [28]. Credibility is defined as trust and confidence in the research findings, and it assesses whether participants’ contributions are correctly interpreted by the researchers [27]. To ensure credibility, interviews were both audio recorded and transcribed using Microsoft Teams™ and transcripts were double checked for accuracy by the research team.

Transferability is defined as the external validity and generalizability of study findings and the extent to which the results can be transferred to other settings and participants [27]. The transferability was ensured through providing detailed description of the study settings and participants’ characteristics and by inviting potential candidates from all 31 PHCC centers for participation [28]. Reflexivity is the researcher’s ability to criticize himself or herself and reflect on his or her own possible biases, preferences, or preconceptions or relationships with the participants and how this may affect the participants’ answers and responses [27]. Reflexivity was attained by researchers disclosing their previous relationships where applicable [28].

### Ethical considerations

Ethical approval for conducting the study was obtained from the Research Department at the PHCC [approval reference number: PHCC/DCR/2020/06/066] and Qatar University Institutional Review Board (IRB) [approval reference: QU-IRB 1458-E/21].

## Results

**Fig 1:**
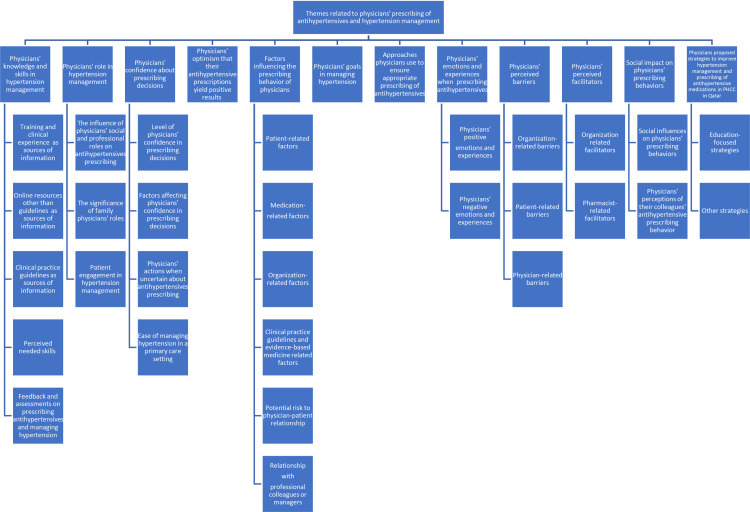
Conceptual diagram of key themes and subthemes.

Thirteen virtual semi-structured one-to-one interviews were conducted between December 2021 and May 2022 (Table 1). Out of 179 eligible participants contacted, 26 participants expressed interest in participating, and eventually, 13 physicians participated in the interviews. Each interview lasted between 45 and 60 minutes. Data saturation was achieved by the 13^th^ interview, indicating no new information emerged thereafter. Twelve themes emerged from the data analysis. The identified themes and subthemes were shared with the study participants interested in the findings. These are illustrated in Fig 1 and summarized below. Relevant quotes are provided in Table 2 to provide context to the results with examples of quotes provided in text.

**Table 1:** Participants’ characteristics.

Participant Code	Gender	Country of origin	Specialty	Years of experience	Years of experience in Qatar	Clinic name
1	Female	Egypt	Family medicine consultant	21	14	Airport Health Center
2	Female	Pakistan	Family medicine consultant	22	6	Rawdat Al Khail Health Center
3	Male	Syria	Family medicine resident	5	4	Qatar University Health Center
4	Female	Ukraine	Family medicine consultant	21	5	Qatar University Health Center
5	Male	Jordan	Family medicine specialist	5	5	Qatar University Health Center
6	Male	Pakistan	Family medicine specialist	15	6	Al Waab Health Center
7	Female	Tunisia	Family medicine resident	5	4	Qatar University Health Center
8	Female	Qatar	Family medicine senior consultant	17	17	Airport Health Center
9	Male	Syria	Internal medicine specialist	25	9	Al thumama Health Center
10	Male	India	General medicine specialist	30	2	Alwakra Health Center
11	Male	India	General medicine specialist	26	13	Alwakra Health Center
12	Male	India	Family medicine + Internal medicine specialist	24	5	Mesaimeer Health Center
13	Male	Pakistan	Family medicine consultant	20	4	Umm ghuwailina Health Center

**Table 2:** Themes, subthemes, and illustrative quotes mapped against the TDF.

Domain	Theme	Subtheme	Quote
Knowledge and skills	Physicians’ knowledge and skills in hypertension management	Training and clinical experience as sources of information	*“My clinical practice and experience are the major resources for my knowledge” P8* *“Actually, I came to know more about hypertension management from the residency program I told you about at Hamad Medical Corporation” P10*
		Online resources other than guidelines as sources of information	*“Through online resources accessible through Google© Chrome we get the latest updates on hypertension management.” P11* *“Yeah, we do online modules. We have subscriptions to several journals including British Medical Journal, so we receive all new information by attending their online modules.” P3*
		Clinical practice guidelines as a source of information	*“Nice guidelines Yeah, so mostly my practice has been to follow the NICE guidelines. The NICE guidelines are the number one guideline that we follow because they are different from the American guidelines, in a way that they are not based on medications’ prices they are just based on their safety.” P5* *“We have PHCC guidelines for management of hypertension. Also, sometimes I’m using it.” P8* *“They send you emails at PHCC to remind you to follow the guidelines that they have especially if there are updates. So, I usually follow them.” P13*
		Perceived needed skills	*“Communicating with the patient is essential because you can inform him/her of the findings and ensure that he/she understands the information that you give him/her. So, it all goes down to communication” P4* *“It is your time management and communication skills which make the difference. You need to possess the necessary skills to ensure that your patients return for follow-up appointments, thus establishing a safety net in the event you initiate therapy, allowing you to monitor their response effectively..” P5* *“We must ask about patients’ history and patients’ family history if they have ischemic heart disease, hypertension, diabetes, hypercholesterolemia, or asthma, heart failure, …etc. In addition, you need to ask the patients about their lifestyle and occupation, and whether they live with their family or not or whether they are facing a problem in their life with their family or friends.” P1*
	Feedback and assessments on prescribing antihypertensives and managing hypertension		*“We don’t get individual feedback on our antihypertensive prescriptions. No, it’s an overall appraisal. We get annual appraisals on how we treat patients in general. We don’t get individual clinical appraisals.” P3* *“In general, we don’t get feedback about antihypertensives, because I think that’s too specific. Occasionally, we get pats on the back that we are doing a good job, that we should carry on, that we should be proud, and if there is anything to change, let’s do a little more. Put more details regarding documentation, but nothing specific to antihypertensive prescribing.” P9* *“PHCCs follow the instructions given by the Ministry of Public Health. Our files are audited. Additionally, we have an audit team, and the audit is conducted on a monthly basis, so they are following our prescribing and sending us reports, so we can determine exactly where we lack in our prescribing-related documentation or in not following guidelines. As a result of these reports, we improve our prescribing.” P7*
Social professional role and identity	Physician’s role in hypertension management	The influence of doctors’ social and professional roles on antihypertensives prescribing	*“Uh, it *doesn't *affect because we prescribe to the patient the best medication that he or she needs to prevent the complications associated with hypertension.” P2* *“Alright, well I don’t think it affects a lot. I just use my own experience and whatever is the prescribing protocol But I don’t think it affects me. I do my job” P13* *“If you’re trying to balance between the two, then you have to see. Uh, the social role is there, but obviously the clinical role takes priority. Ah. So, the clinical knowledge will take the priority and the clinical need will take the priority” P9*
		The significance of family physicians’ roles	*“The family physician is the one who knows the patient the most you know it’s an important specialty especially in hypertension management because the family physician is the only one who can counsel the patient very well. He can do regular follow ups with the patient.” P10* *“So, for us primary care physicians, we are the basic block in identifying hypertensive patients, diagnosing them, and initiating treatment.” P3* *“So, it is as I said it earlier it is not only about prescribing and scheduling periodic visits for refills every two months. Hypertension, being a chronic condition, necessitates thorough and continuous monitoring, similar to the approach taken for managing diabetes.” P2*
		Patient engagement in hypertension management	*“However, we must always come to an agreement, otherwise the patient will not take any prescribed medication. In order to provide the best care for the patient, we have to find out what’s best for him/her? Afterwards, we’ll come to an agreement between us after I explain it to him or her” P4* *“. It is the clinician’s job to come to a consensus after discussing and explaining all options.” P5*
Beliefs about capabilities	Physicians’ confidence about prescribing decisions	Level of physicians’ confidence in prescribing decisions	*“I should say strongly confident” P5* *“I think I’m confident 100%, I practice it and I read about it” P11* *“Reasonably confident, I suppose.” P9*
		Factors that affect physicians’ confidence in prescribing decisions	*“Experience of 15 years is a long time, so I think that’s probably the main source of confidence.” P4* *“Your confidence comes from your knowledge.” P5* *“And what makes me reasonably confident? It’s basically the guidelines that we usually follow” P9* *“I am following up with my patients after two weeks. They come back with their hypertension readings. So, I can ensure the medications are really effective. That’s why I become confident when prescribing.” P10* *“Because what I usually do is to read the patient medical file before he or she enters my office.” P8*
		Physicians’ actions in case of uncertainty about antihypertensives prescribing	*“Yes, one of course we are facing some difficult cases which we are like. Uh, not sure if this medicine is suitable for the patient or not, I usually take a second opinion from my colleague. So, I ask my colleague who is either a consultant or a senior consultant, uh, what do you think about this case?” P7* *“Yeah. OK. So, in this case, I’ll give him, to be honest a medication according to the guidelines. My next step is to follow up with him after one week, not after two weeks, so I can make sure the medication is appropriate for this patient.” P10* *“The clinical pharmacists we have in the health center can sometimes guide us and support us with medication-related information” P8*
		Ease of managing hypertension in a primary care setting	*“OK, it is much harder to manage patients who have multiple comorbidities and are taking medications for these comorbidities than somebody who has only been hypertensive. It’s straightforward for patients with simple hypertension” P3* *“Whether being a specialist or consultant at PHCC we are all able to manage hypertensive patients unless there are some cases which are not very common where the hypertension cannot be managed at PHCC in this case we refer patients to the secondary care. We know when referral is needed, but otherwise we are dealing with hypertension management here at PHCC.” P7* *“So, I suppose it is a combination of factors, the guidelines, the practical experience you have. Obviously prescribing antihypertensives becomes easy with time.” P9*
Optimism	Physicians’ optimism that their antihypertensive prescriptions yield positive results		*“I am confident that my prescribing of antihypertensives will yield positive outcomes eight out of 10.” P10* *“I am confident that my prescribing will yield positive outcomes Let’s say 8.” P11*
Beliefs about consequences	Factors influencing the prescribing behavior of physicians	Patient-related factors	*“Prescribing of antihypertensives depends on the patient’s age” P12* *“Prescribing of antihypertensives depends on the ethnicity of the patient” P8* *“The major things for the treatment of hypertension are not related to the medication, they are related to the nonpharmacological treatment including lifestyle changes.” P10* *“I mean so you know the financial status also is a very important factor.” P6* *“Gender, gender is the main factor that influences the prescribing of antihypertensives. It is not only about just prescribing and dispensing the pill; the treatment should be patient-centered and the patient should be engaged. I think about patient potential compliance to my prescribed drug regimen.” P3* *“Uh, if they have any other chronic diseases like diabetes. This will affect my prescribing. As we always teach the medical students as well. you need to adopt a holistic approach for managing hypertension. You need to look at the patient as a whole. Their medication compliance is the main factor that influences my prescribing” P5*
		Medication-related factors	*“As a family physician my role is primary prevention of cardiovascular diseases, so I must calculate the risk versus benefits of antihypertensives.” P5* *“If a medicine harms the patient. ACE inhibitors, for example, can cause dry cough, which can be really irritating, so we need to switch the patient to another medication.” P2* *“OK, I can change my decision depending upon the medication side effects” P9*
		Organization-related factors	*“The first thing is the availability of a particular medication in our pharmacy. I cannot prescribe a medication that is not available.” P13* *“Another factor that impacts my decision is the availability of the medicine in our formulary at the clinic” P7*
		Clinical practice guidelines and evidence-based medicine related factors	*“I follow NICE guidelines, that are evidence based.” P5* *“To begin with, I will refer to the guidelines that I have the most experience with, as that is what determines my prescribing decisions and as they are evidence-based” P2* *“Also, the patient’s tolerability. It is sometimes difficult for the patient to tolerate a medicine recommended by the guidelines. For example, this medicine is the first line and a great medicine for this patient, given his age and situation, but he is unable to tolerate it.” P7*
		Potential risk to physician-patient relationships	*“If you are explaining the benefits, disadvantages and side effects of the medication to the patient clearly and there is mutual understanding between the patient and the physician, the relationship between both will not be affected. Therefore, I make my prescribing decisions regardless of whether it affects my relationship with the patient” P6* *“Because I generally explain to my patients why I prescribe this medication, if they ask why it is not the right medication or why it is the right medication, I explain the reasons and it does not affect our relationship..” P13* *“It is my responsibility to make a decision based on the patient’s signs and if it is the right choice, not based on my relationship with that patient.” P5*
		Relationship with professional colleagues or managers	*“Not much, I don’t think anyone from my managers or colleagues interfere with my prescribing.” P8* *“I don’t think at PHCC there are any issues regarding the availability of medications. Sometimes we get a circular to prescribe the medications that are near expiry this is the only time you feel you are forced to prescribe specific antihypertensives.” P13* *“I did not experience this, but some colleagues believe in one medication or company. We are expected to prescribe medications from a specific company, and we are expected to prescribe like them. Several colleagues prescribe the same medication regardless of patient condition, which does not align with patient safety. If we prescribe against what the medical rep wants, we will lose our relationship with these colleagues.P7* *“No, I haven’t observed this happening because, like everyone else, I’m focused on practicing and doing my job. It doesn’t impact my relationship with colleagues, and it won’t influence my decisions. However, it might affect other doctors. For me, my decisions remain unaffected; I won’t prescribe until I thoroughly review all information about the medication, its efficacy in treating hypertension, and potential side effects, regardless of a medical representative’s input..” P10*
Goals	Physicians’ goals in managing hypertension		*“Achieving blood pressure control is my goal.” P1* *“My goal is to prevent elevated blood pressure complications..” P11* *“My goal is to make sure that the patient is compliant with his or her medications, and there’s no or minimal side effects. And then I want to engage my patients.” P5* *“My goal is to use the least effective dose with the least side effects.” P12* *“OK, my first goal is that the patient should understand his or her condition” P8*
Behavioral regulation	Approaches physicians use to ensure appropriate prescribing of antihypertensives		*“In order to ensure that my antihypertensive choice was appropriate I follow-up with the patient and monitor his or her blood pressure.” P8* *“During the follow up, we will see how the patient is doing and how his BP is responding.” P12* *“As I mentioned before, I keep myself up to date with the latest guidelines. Staying up to date is the best way to ensure your prescribing decisions are appropriate.” P5*
Emotions	Physicians’ emotions and experiences and their effect on prescribing	Physicians’ positive emotions and experiences when prescribing antihypertensives	*“It depends on the patient. If it’s just uh, for example, if the patient is newly diagnosed and if I just prescribe the medication for the first time, I will have positive emotions. I’m so happy.” P12* *“Positive, always positive. So, whenever a doctor prescribes a medication to help his or her patient this is considered a positive experience and I will always feel positive about it.” P2* *“But it looks like that every day we prescribe a medication, if the patient comes back to me and his blood pressure is better than before. Then that is a positive experience So it’s kind of a satisfying experience that you can help somebody and change his or her life.” P5* *“Yeah, I will share you with you one of my patients was taking an ACEI with a CCB for his hypertension and after a while he developed lower limb edema and while discussing the case with him we thought that the cause was the ACEI with the CCB so we changed the blood pressure medication from ACEI inhibitor to a thiazide diuretic and within a week, the edema was resolved. So, this was a great experience for me with one of the difficult patients.” P11* *“It was a positive experience for both physicians and patients.” P13*
		Physicians’ negative emotions and experiences when prescribing antihypertensives	*“There are times when I am stressed when I see the blood pressure log is not controlled or when hypertensive patients come in with high blood pressure. Medications’ side effects also can make it a negative experience when prescribing antihypertensives” P3* *“I feel concerned as I told you about the patient if his blood sugar or BP are not controlled well with my prescribed medication, and he should give me feedback. Good feedback.” P13* *“Let’s say you have a patient with hypertension who wants a particular medicine. Despite telling you about it, he doesn’t know much about it; nor does he know its name; so, he asks for it, and he can’t tell you where he got it or what the medicine is or how much he took. You might feel anxious in such a situation.” P9* *“As an example, a patient who was already on an ACEI was coming to me with chronic cough, and I had to stop him from taking the medication. I explained to her that ACEI caused the cough, and sometimes patients take the medication so badly that a side effect does not wear off easily. As a result, I had to switch it to an ARB, so yeah, in a sense, it was a negative experience.” P12* *“Negative experiences. Mmmmmm for example, if a patient is not compliant with the medication, this is a negative experience for me.” P2*
Sociopolitical and organizational context and resources	Physicians perceived barriers when prescribing antihypertensives and managing hypertension	Organization-related barriers	*“OK, the first barrier and this is very common when you do not have the medication in the pharmacy of the health center” P10* *“And also, the secondary care like is there somebody on call we can speak to? We don’t have that access here at all, so there is no communication between us and the secondary care. I’m used to writing letters and the consultant used to write us back. So, there’s no communication between primary and secondary care which is the biggest gap here in patient management.” P3* *“Sometimes also system based, like the Cerner© for example, it can be slow, so it takes time. Sometimes it blocks completely.” P12*
		Patient-related barriers	*“The second thing is like the cost of the medication is also affecting our prescribing of medications to some patients, you know some of our patients have low salaries and they cannot afford paying for their medications. Another barrier is the side effect of the medication some patients cannot tolerate the side effects of certain medications.” P10* *“Well, if you talk about it, you see that there are some financial barriers as there are patients who are unable to afford their medications, but PHCC subsidizes medications at a low price.” P5* *“Language is another probably important barrier because sometimes patients don’t understand what you’re talking about.” P4*
		Physician-related barriers	*“time is very limited in our consultations this can be a barrier. But we try to figure that out.” P11* *“Whenever I see a patient with poorly controlled blood pressure, and I don’t have the time to sit with him or her I refer him or her to the NCD clinics.” P5* *“It’s really, really hard to attend sometimes because it’s your personal time or it’s the patient’s time. Uh, there’s no protected time.” P9*
	Physicians perceived facilitators when prescribing antihypertensives and managing hypertension	Organization-related facilitators	*“The availability of the most important medications is a facilitator.” P11* *“Yeah, we have so many resources here that I’m always using such as UpToDate®.” P8* *“OK, so first of all Cerner® Like it is really a good system. So, it sends us an alarm if there is a side effect or a contraindication or if the patient is taking another medication that interacts with the prescribed medication.” P10* *“If you take this computer away from me, then my prescribing decision will not be as safe.” P5* *“PHCC is a very supportive environment.” P9* *“We can download the courses and the workshops that we need and then there will be like a quiz that we need to pass. So, you get to be knowledgeable from the scientific point of view and this is how the organization supports us.” P7*
		Pharmacist-related facilitators	*“No, I suppose I was about to say that yes, if I have any queries, I quite freely call my pharmacy colleagues. And I speak to them with regards to medications and I take advice from them as well.” P9* *“There’s a safety net when communicating with the pharmacist.” P5* *“And for example, this medication *shouldn't *be prescribed with another medication, and then we can modify and change our prescribed medications” P8*
Social influences	Social impact on physicians’ prescribing behaviors	Social influences on physicians’ prescribing behaviors	*“There is no pressure from patients or families.” P2* *“No, I don’t have to. If there is a clinical need, then of course that can be discussed. But if there is no clinical need, I won’t prescribe the medication requested by the patient.” P9* *“I’m quite insisting. I will do what’s right for the patient, not what the patient wants to do.” P4* *“Therefore, if I feel that the medication will harm the patient or if I know from studies that it will harm the patient, I will not prescribe it. Instead, I will provide the patient with an alternative medication that won’t cause harm.” P1* *“I think that some patients originating from the Middle East are very much geared towards the brand name of bisoprolol, and it isn’t the first line of treatment. As I’m talking to you, I just remembered meeting some patients prematurely prescribed this medication in private clinics and they ended up suffering from harm. There were a couple of times when I found it very difficult to convince my patients otherwise.” P4* *“If the spouse is taking a medication, then they may ask why they are taking a different one. However, we discuss with them the pros and cons of the medication that they want versus the one we recommend.” P13* *“Yeah, no that is true for general practice because some of our patients travel outside Qatar. They get their medications from abroad and they’re happy with it since they don’t suffer from any side effects, so they want to continue using it.” P9*
		Physicians’ perceptions of their colleagues’ antihypertensive prescribing behavior.	*“Generally, we practice in the same lines and because we are all trained in the same way.” P5* *“So, it’s not a major difference because it’s the same medication. I prescribe ABCD, and my colleague might prescribe DEFGH. But it’s not wrong at the end of the day, because it’s part of the guidelines. OK, it’s a different individual practice and we may be different from each other, but we don’t criticize each other.” P3* *“It is normal to not always agree with my colleague’s prescription because everyone has different opinions. Sometimes other physicians won’t accept what you prescribe, and sometimes I won’t accept what they prescribe.” P1* *“Here, we have a very open culture, which creates a lot of trust. When there is a problem regarding a patient’s case, we can discuss it as a group and admit our mistakes with some patients, ensure patient safety, and discuss everything else, so it’s okay.” P4*
Strategies	Physicians proposed strategies to improve hypertension management and prescribing of antihypertensive medications in PHCC in Qatar	Education-focused strategies	*“More education, more training will help a lot.” P2* *“In fact, if there had been many workshops or something like that, it would have been great, but because of this COVID-19 pandemic, there have been fewer workshops and CMEs” P13* *“Student precepting keeps us updated and gives us reminders, for example, to access the LMS and read guidelines. Our knowledge can generally be improved by having a protected time. In the future, we could give physicians a protected time to read guidelines, or the latest articles.” P8*
		Other strategies	*“Improving Cerner® I think all doctors will agree on that.” P12* *“Expanding the PHCC formulary list as some medications are not available so we hope that they will make them available in our practice.” P7* *“If they brought back noncommunicable disease (NCD) clinics it would be more beneficial for patients. Believe me back when noncommunicable disease (NCD) clinics were available our patients’ BP and blood sugar were more controlled. The percentage of controlled patients were about more than 60% or 65%, however, now after they closed these clinics the percentage became less than 50% you know why because now most patients come, and find the physicians very busy” P1* *“It is a good strategy to involve us in chronic patient management since secondary care is overburdened. The acute complicated cases should be transferred to secondary care without any hesitancy. Moreover, we need fast appointments rather than late ones. Follow ups appointments were better in NCDs.” P3* *“I would suggest having some sort of standardizing strategy for all physicians to be prescribing the same class of medications.” P9* *“Education is important for patients too. They should be educated that they have to wait for their number and their turn they cannot just enter the physician room distracting the physician and breaching the confidentiality of other patients.” P5*

**TDF domain: Knowledge and skills**

**Theme 1: Physicians’ knowledge and skills in hypertension management**

**Subtheme 1a: Training and clinical practice as sources of information**

Several respondents considered their residency training and clinical experience as primary sources of knowledge.

*“My clinical practice and experience are the major resources for my knowledge”* ***P8***

**Subtheme 1b: Online resources other than guidelines as sources of information**

Others, however, cited using literature searches or online resources other than guidelines such as search engines like Google ^©^ or others as sources of information.

*“Through online resources accessible through Google© we get the latest updates on hypertension management.”* ***P11***

**Subtheme 1c: Clinical practice guidelines as sources of information**

Many respondents identified clinical practice guidelines as their primary information source for hypertension management, with the majority stating reliance on one or more guidelines. These included national guidelines like the PHCC guidelines, as well as international guidelines such as NICE guidelines, JNC 8, and American Heart Association (AHA) guidelines, among others. Furthermore, several interviewees emphasized the importance of staying current with guidelines and regularly checking for updates. Some physicians also reported receiving reminders about updates to the guidelines from the PHCC administration, which they found beneficial for staying informed.

*“We have PHCC guidelines for the management of hypertension. Also, sometimes I’m using it.”* ***P8***

**Subtheme 1d:** **Perceived needed skills**

Nearly all physicians agreed that communication skills are essential when prescribing antihypertensives and managing patients with hypertension. It was also noted by some physicians that proper history-taking skills, the ability to convince the patient to attend follow-up appointments, effective time management skills, and self-confidence were all important skills in building patient trust.

*“It is your time management and communication skills which make the difference. You need to possess the necessary skills to ensure that your patients return for follow-up appointments, thus establishing a safety net in the event you initiate therapy, allowing you to monitor their response effectively.”* ***P5***

**Subtheme 1e: Feedback and assessments on prescribing antihypertensives and managing hypertension**

The participants provided varied responses regarding the feedback they received about their prescribing practices. Some physicians stated they had not received any specific feedback or assessments concerning their antihypertensives prescribing. On the other hand, others mentioned receiving general feedback that was not specifically focused on their antihypertensives prescribing. A few physicians noted that that their health centers’ administration conducts quality checks specifically on their antihypertensives prescribing practices.

*“We don’t get individual feedback on our antihypertensive prescriptions. No, it’s an overall appraisal. We get annual appraisals on how we treat patients in general. We don’t get individual clinical appraisals.”* ***P3***

**TDF domain: Professional role and identity**

**Theme 2: Physician’s role in hypertension management**

**Subtheme 2a: The influence of doctors’ social and professional roles on antihypertensives prescribing**

Many participants stated that their prescribing decisions for antihypertensive medications are not influenced by their social or professional roles. They emphasized that their clinical roles take precedence over any social considerations.

*“Alright, well I don’t think it affects a lot. I just use my own experience and whatever is the prescribing protocol, but I don’t think it affects me. I do my job”* ***P13***

**Subtheme 2b: The significance of family physicians’ roles**

Several physicians highlighted the crucial importance of the family physician’s role in diagnosing and managing hypertension Furthermore, many physicians emphasized that they believe family physicians play several crucial roles in the treatment of hypertensive patients, including educating patients, providing counseling, and offering advice.

*“So, for us primary care physicians, we are the basic block in identifying hypertensive patients, diagnosing them, and initiating treatment.”* ***P3***

**Subtheme 2c: Patient engagement in hypertension management**

Several respondents noted that patients tend to adhere more to their treatment plans when they are actively engaged in their own treatment plans. They stressed the importance for physicians to collaborate with patients in reaching agreements on their pharmacotherapy regimen.

*“However, we must always come to an agreement, otherwise the patient will not take any prescribed medication. In order to provide the best care for the patient, we have to find out what’s best for him/her. Afterward, we’ll come to an agreement between us after I explain it to him or her.”* ***P4***

**TDF domain: Beliefs about capabilities**

**Theme 3: Physicians’ confidence about prescribing decisions**

**Subtheme 3a: Level of physicians’ confidence in prescribing decisions**

When physicians were asked about their confidence in prescribing antihypertensive medications, the majority answered that they were highly confident in their prescribing decisions.

*“I think I’m confident 100%, I practice it and I read about it.”* ***P11***

**Subtheme 3b: Factors affecting physicians’ confidence in prescribing decisions**

Many physicians stated that various factors influence their confidence in prescribing decisions such as knowledge, experience, adherence to guidelines, and patient follow-up. Other physicians mentioned additional factors such as educating patients about medication side effects, listening to their concerns, and reviewing their medical profiles.

*“Experience of 15 years is a long time, so I think that’s probably the main source of confidence.”* ***P4***

**Subtheme 3c: Physicians’ actions when uncertain about antihypertensives prescribing**

The majority of the participants indicated that when they encounter uncertainties in prescribing antihypertensives, they typically look for information in medical resources or seek advice from a senior colleague. Other participants mentioned strategies such as scheduling more frequent follow-ups with the patient, consulting available pharmacists, and prescribing standard antihypertensive agents while ensuring the patient has no contraindications.

*“The clinical pharmacists we have in the health center can sometimes guide us and support us with medication-related information”* ***P8***

**Subtheme 3d: Ease of managing hypertension in a primary care setting**

The majority of respondents considered hypertension management as straightforward, especially handling simple uncomplicated cases within a primary care setting. However, some participants emphasized that patients with complicated hypertension may require referral to specialists and cannot be effectively managed solely in a primary care setting. Moreover, when physicians were asked about what makes hypertension management easy, some cited experience and familiarity with guidelines as the main factors for making hypertension management easy.

*“OK, it is much harder to manage patients who have multiple comorbidities and are taking medications for these comorbidities than somebody who has only been hypertensive. It’s straightforward for patients with simple hypertension”* ***P3***

**TDF domain: Optimism**

**Theme 4: Physicians’ optimism that their antihypertensive prescriptions yield positive results**

All of the participating physicians expressed optimism that their prescribing of antihypertensive medications would yield positive outcomes such as improved blood pressure control, and the prevention of complications like stroke and myocardial infarction.

*“I am confident that my prescribing of antihypertensives will yield positive outcomes eight out of 10.”* ***P10***

**TDF domain: Beliefs about consequences**

**Theme 5: Factors influencing the prescribing behavior of physicians**

**Subtheme 5a: Patient-related factors**

Many respondents highlighted that patients’ medical and medication history including comorbidities and chronic medications, as well as age, and ethnicity, are the main factors that significantly influence their decisions when prescribing antihypertensive medications. Some physicians also mentioned patients’ gender, the stage of hypertension, lifestyle factors, medication adherence, and financial status as additional factors affecting their prescribing behavior.

*“Prescribing of antihypertensives depends on the patient’s age.”* ***P12***

**Subtheme 5b:** **Medication-related factors**

Respondents generally agreed that the potential benefits and risks of medications influence their prescribing decisions when treating hypertension.

*“OK, I can change my decision depending upon the medication side effects”* ***P9***

**Subtheme 5c:** **Organization-related factors**

Participants stated that the availability of medications in the drug formulary at PHCC also plays a role in their decisions regarding prescribing antihypertensive medications.

*“Another factor that impacts my decision is the availability of the medicine in our formulary at the clinic”* ***P7***

**Subtheme 5d:** **Clinical practice guidelines and evidence-based medicine-related factors**

The interviewees indicated that their prescribing decisions are primarily based on clinical practice guidelines and evidence-based medicine. However, a few respondents noted that while medications may be strongly supported by evidence and recommended by guidelines, they may not always be optimal for every patient.

*“I follow NICE guidelines, that are evidence-based.”* ***P5***

**Subtheme 5e:** **Potential risk to physician-patient relationships**

Most physicians believed that their prescribing decisions do not harm their relationships with patients, especially when they provide explanations about the benefits and possible side effects of the medications and offer follow-up care.

*“It is my responsibility to make a decision based on the patient’s signs and if it is the right choice, not based on my relationship with that patient.”* ***P5***

**Subtheme 5f:** **Relationship with professional colleagues or managers**

According to most participants, their professional relationships with colleagues or managers have affected how they select antihypertensive medications or make prescribing decisions. Yet, some participants suggested that medical representatives could influence physicians’ prescribing behaviors, potentially affecting their relationships with colleagues who oppose such practices.

*“Not much, I don’t think anyone from my managers or colleagues interfere with my prescribing.”* ***P8***

**TDF domain: Goals**

**Theme 6: Physicians’ goals in managing hypertension**

Most interviewees emphasized that their main goals in managing hypertension include achieving BP targets and reducing and preventing hypertension-related complications. While others declared their goals include ensuring medication compliance with minimal side effects and prescribing the lowest effective medication dose. Moreover, a few physicians highlighted patient engagement as an important goal in hypertension management, as patients need to understand well their condition.

### “Achieving blood pressure control is my goal.” P1

**TDF domain: Behavioral regulation**

**Theme 7: Approaches physicians use to ensure appropriate prescribing of antihypertensives**

When participants were asked about how they ensure the appropriateness of their antihypertensive medications prescribing, the majority indicated that regular follow-ups with patients are key. They also noted that improving patients’ outcomes, along with achieving target blood pressure levels are indicators of optimal use of antihypertensives.

*“In order to ensure that my antihypertensive choice was appropriate I follow-up with the patient and monitor his or her blood pressure.”* ***P8***

**TDF domain: Emotions**

**Theme 8: Physicians’ emotions and experiences when prescribing antihypertensives**

**Subtheme 8a: Physicians’ positive emotions and experiences when prescribing antihypertensives**

For some physicians, prescribing antihypertensives to patients was a positive experience, especially when the patient’s BP was controlled. In addition, a few physicians felt positive when patients returned for their follow-up appointments.

*“Positive, always positive. So, whenever a doctor prescribes a medication to help his or her patient this is considered a positive experience and I will always feel positive about it.”* ***P2***

**Subtheme 8b: Physicians’ negative emotions and experiences when prescribing antihypertensives**

Most interviewed physicians considered prescribing medications that proved ineffective or caused side effects for their patients as a negative experience. As an example, some physicians found prescribing ACEIs to be a negative encounter when patients developed coughs as a side effect. Conversely, others considered it an unfavorable experience when the patient did not adhere to their prescribed medications. Additionally, some physicians reported feeling stressed and concerned when their patients’ blood pressure is not controlled.

*“There are times when I am stressed when I see the blood pressure log is not controlled or when hypertensive patients come in with high blood pressure. Medications’ side effects also can make it a negative experience when prescribing antihypertensives”* ***P3***

**TDF domain: Environmental context and resources**

**Theme 9:** **Physicians perceived barriers when prescribing antihypertensive medications and managing hypertension**

**Subtheme 9a: Organization-related barriers**

Several organizational barriers were perceived by interviewees, such as medication shortages, limited medications in the PHCC formulary, and inappropriate BP measurement conditions. Additionally, some physicians perceived that there was insufficient patient-physician interaction outside of clinic settings, that patients did not always return for follow-up appointments with the same physician, and that there was a lack of communication between primary and secondary care in the country. Moreover, some physicians reported occasional technical errors in the Cerner^©^ electronic medical record system and delays in referring patients to secondary care.

*“OK, the first barrier, and this is very common when you do not have the medication in the pharmacy of the health center”* ***P10***

**Subtheme 9b: Patient-related barriers**

Physicians interviewed identified several patient-related barriers to prescribing antihypertensive medications. These barriers included concerns about medication costs and side effects, language barriers, as well as issues related to patients’ noncompliance.

*“Language is another probably important barrier because sometimes patients don’t understand what you’re talking about.”* ***P4***

**Subtheme 9c: Physician-related barriers**

Interviewees noted that they had limited time for consultations with patients, and therefore referred patients to NCD (Non-Communicable Diseases) clinics to overcome this limitation. Moreover, some physicians indicated a lack of time to attend continuing education webinars.

*“Time is very limited in our consultations this can be a barrier. But we try to figure that out.”* ***P11***

**Theme 10:** **Physicians perceived facilitators when prescribing antihypertensive medications and managing hypertension**

**Subtheme 10a: Organization-related facilitators**

Most interviewed physicians viewed available resources as facilitators, including online drug information resources, and PHCC educational webinars. A number of physicians also pointed out that Cerner’s^®^ medical record system played a critical role in ensuring safe medication prescribing practices.

*“The availability of the most important medications is a facilitator.”* ***P11***

**Subtheme 10b: Pharmacist-related facilitators**

Most physicians acknowledged that pharmacists’ availability is a major facilitator for antihypertensive prescribing and hypertension management at PHCCs.

*“There’s a safety net when communicating with the pharmacist.”* ***P5***

**TDF domain: Social influences**

**Theme 11: Social impact on physicians’ prescribing behaviors**

**Subtheme 11a: Social influences on physicians’ prescribing behaviors**

In general, respondents reported that they do not let patients, caregivers, or families influence their decisions when prescribing antihypertensives. They emphasized that they would not prescribe medications that are not clinically indicated or could potentially harm their patients. For them, patient safety comes first when managing hypertension. However, several physicians admitted feeling pressured sometimes to prescribe specific antihypertensive medications when patients are resistant to their advice and are difficult to convince. According to these physicians, this problem stems partly from the prescribing practices of certain private sector physicians in Qatar or from patients obtaining these medications from abroad.

*“There is no pressure from patients or families.”* ***P2***

**Subtheme 11b:** **Physicians’ perceptions of their colleagues’ antihypertensive prescribing behavior**

Overall, the interviewed physicians believed that their colleagues prescribe antihypertensive medications similarly due to shared training background and adherence to guidelines. Moreover, PHCC fosters an open discussion culture where physicians discuss patients’ cases with their colleagues.

*“Generally, we practice along the same lines because we are all trained in the same way.”* ***P5***

**Strategies (not related to TDF)**

**Theme 12: Physicians proposed strategies to improve hypertension management and prescribing of antihypertensive medications in PHCC in Qatar**

**Subtheme 12a: Education-focused strategies**

The interviewed physicians proposed several approaches to improve the prescribing of antihypertensive medications and the management of hypertension within the PHCC setting in Qatar. The suggestions included educating nurses and physicians, organizing conferences and workshops, reminding physicians to regularly consult guidelines when prescribing, and precepting students to stay updated. Moreover, the physicians suggested allocating protected time for updating their knowledge and reviewing guidelines and literature.

*“More education, more training will help a lot.”* ***P2***

**Subtheme 12b: Other strategies**

The interviewees further proposed other strategies to improve the management of hypertension and prescribing of antihypertensive medications in primary care settings. These include improving home BP monitoring, optimizing the use and functions of Cerner^®^, adding more medications to the formulary, re-establishing the NCD clinics, emphasizing the role of primary care physicians to reduce secondary care burden, increasing consultation times, and standardizing antihypertensives prescribing practices among physicians. Other proposed strategies included establishing support groups for patients with hypertension to improve adherence and educating patients on proper etiquette when entering physicians’ rooms.

*“If they brought back noncommunicable disease (NCD) clinics it would be more beneficial for patients. Believe me back when noncommunicable disease (NCD) clinics were available our patients’ BP and blood sugar were more controlled. The percentage of controlled patients was about more than 60% or 65%, however, now after they closed these clinics the percentage became less than 50% you know why because now most patients come, and find the physicians very busy”* ***P1***

## Discussion

This study investigated the perceptions and experiences of primary care physicians in Qatar regarding hypertension management. It also explored their prescribing behavior as well as their perceived factors for the effective management of hypertension in primary care settings. These participants identified several resources that contributed to their hypertension-related knowledge such as experience, and clinical practice guidelines. These resources also function as a source of information for managing various diseases besides hypertension. A 2018 review of 33 studies revealed that clinical practice guidelines serve as a significant source of knowledge for physicians and influence their medication prescribing decisions [29]. Furthermore, a systematic review of 19 studies found that pocket-based antibiotic guidelines were judged to be the most helpful source of information for prescribing antibiotics [30].

The study results showed that the physicians’ self-confidence in managing hypertension was generally high. These results are in line with a 2023 study that evaluated the practices, attitudes, and expertise of primary care physicians in Qatar. According to this study, primary care physicians in Qatar were highly confident in their ability to treat hypertension, with the majority adhering to both local and international clinical practice guidelines [31]. Furthermore, a strong association was observed between physicians’ confidence level and the frequency of treating hypertensive patients; physicians who managed hypertensive patients daily exhibited greater confidence compared to those who treated patients less frequently, highlighting the impact of experience in nurturing self-confidence in hypertension management [31]. The high self-esteem observed among interviewed primary care physicians in Qatar is reassuring as it can improve their therapeutic decision making and strengthen patient trust ultimately leading to better clinical and humanistic outcomes for patients with hypertension [32]. Moreover, physicians’ self-confidence can enhance their ability to manage stress and to elevate their mood which is essential given the relatively high percentage of burnout among healthcare professionals in Qatar [33, 34].The interviewees also emphasized their role as family physicians which extends beyond prescribing medications. It includes educating and advising patients to ensure their engagement in hypertension management. Similarly, in a qualitative study conducted in the United Kingdom (UK) using the TDF, general practitioners (GPs) perceived their professional role and identity as facilitators in achieving BP targets [35].

The physicians who were interviewed highlighted several factors that affect their prescribing behavior such as those related to patients, organizations, medications, and evidence-based medicine. As recommended by hypertension management guidelines patient-related factors should be taken into account when prescribing antihypertensive medications [6–8].

Moreover, most interviewed physicians mentioned medications’ side effect profiles among the factors that influenced their prescribing of certain antihypertensives and these findings are also supported by the published literature [36]. Researchers in Greece and Cyprus found similar results, with 90% of physicians changing their prescribing patterns because of medications’ side effects [37]. Furthermore, physicians reported following hypertension management guidelines and evidence-based medicine which aligns well with published studies elsewhere [38].

Physicians’ adherence to guidelines was assessed in several published studies in the literature with one study assessing physicians’ role in hypertension management and their compliance to JNC7, World Health Organization/International Society of Hypertension (WHO/ISH), and European Society of Hypertension (ESH) guidelines in Nigeria [39]. The study revealed that physicians followed the recommendations for hypertension management [39]. Conversely, a Malaysian study discovered that physicians did not follow clinical practice standards, and that when physicians followed recommendations for managing hypertension, hypertension control improved [40]. The adherence of primary care physicians in Qatar to clinical practice guidelines as compared to other regions reflects the exceptional quality control measures implemented within the Qatari healthcare system specifically in the primary care system. These measures are mandated by the government and driven by Qatar Healthcare Strategy as well as recommended by the accreditation and certification bodies of the PHCC in Qatar [41, 42].

Physicians’ patient-oriented goals were an important finding throughout the interviews. This finding is very encouraging as embracing patient-oriented goals has the potential to improve patient outcomes especially in achieving optimal BP control and ensuring better translation of clinical trial findings into real-life clinical practice. For instance, BP control was assessed in 437 patients who underwent follow-up for at least one year at a specialist clinic implementing “goal-oriented management” in 2002 in the United States. According to the study, goal-oriented management significantly improved control rates over standard practice, demonstrating its usefulness in applying clinical trial outcomes to outpatient care [43].

Interviewed physicians identified certain resources within the PHCC organization as barriers. These obstacles are similar to those reported in previous studies about physicians’ challenges in managing chronic conditions [44, 45]. Furthermore, interviewed physicians reported some perceived patient-related barriers that included medications’ price, side effects, language barriers, and patients’ noncompliance. In comparison, a systematic review conducted in 2021 to assess the factors related to inappropriate prescribing and barriers for medication optimization among elderly patients in the primary care settings published similar results regarding patients’ related barriers including patient response to side effects [46–49]. Qatar’s primary healthcare centers currently subsidize medications’ prices, making them more affordable for patients. However, it would be beneficial to assess further lowering of medication prices for patients with relatively low salaries. Proposed strategies to enhance medication adherence include following up with patients through the reestablishment of NCD clinics and recruiting multilingual staff to explain the importance of adhering to prescribed medication regimens to patients facing communication barriers.

Conversely, the interviewees highlighted some facilitators for prescribing antihypertensives including organization-related facilitators such as availability of information resources and supporting software. In line with the results of numerous studies conducted elsewhere, several interviewees also considered the presence of pharmacists as a facilitator in their prescribing of antihypertensives.[49, 50]. Physicians’ recognition of pharmacist’s role in hypertension management is promising given the vital contribution of pharmacists to Qatar’s governmental healthcare sector. Pharmacy practice in Qatar has greatly advanced in the past decade transitioning from traditional medication dispensing to offer holistic patient centered care [51].

On the other hand, social influences were not identified as major influencing factors for physicians’ prescribing behavior. Most physicians indicated that their decisions were not affected by patients ‘requests and that they prescribed what was appropriate for their patients. They suggested that proper patient education improves compliance with prescribed therapy. In comparison, the social influences of patients were deemed as important behavioral determinants in the literature. For instance, in 2018, Van Middeaar et al. published a study evaluating prescribing and deprescribing of antihypertensives in elderly patients in the Netherlands using semi-structured interviews and found that patients’ requests influenced both their prescribing and deprescribing behaviors [52]. Several published studies also revealed that patients’ preferences played a significant role in physicians’ prescribing decisions [30,53–59]. In this study, interviewed physicians reported feeling uncomfortable whenever patients insisted on using specific medications similar to the findings in a 2011 study conducted in the UK [60]. Several factors can explain the results of the Qatari study. Primary care physicians in Qatar largely rely on clinical practice guidelines for their prescribing decisions with the goal of selecting the most appropriate treatment for patients. Another potential factor is the time constraint as it may prevent physicians to have the needed time to consider patients’ preferences. In fact, in a previous survey 75% of primary care physicians identified lack of time for consultation as barrier for hypertension management [31]. Moreover, the availability of medications in the PHCC formulary may hinder physicians from accommodating patients’ requests.

Notably, a theme related to the feedback and assessments that physicians received regarding their prescribing of antihypertensives emerged throughout the interviews. Based on these results, PHCC clinics should implement a standardized system utilizing active strategies with audit and continuous feedback to physicians about their prescribing decisions for managing chronic diseases, including hypertension [61].

Several strategies were recommended by participants to improve hypertension management in Qatar, including patients monitoring their blood pressure at home, this has been proven to be a highly effective strategy for blood pressure control in hypertensive patients [62, 63]. Other recommended strategies included Cerner^®^ system optimization, adding more medications to the PHCC formulary, and re-establishing NCD clinics. NCD clinics are effective in hypertension management as demonstrated in a study conducted in Rwanda in 2022 [64]. This study revealed that patients treated for either diabetes or hypertension or both in NCD clinics have a higher level of hypertension control than patients managed in family practice clinics [64]. Previously PHCC had NCD clinics and further investigation is needed to assess the logistical and financial feasibility of reintroducing them.

### Strengths and limitations

This study has several strengths. This is the first study in Qatar to assess primary care physicians’ perceptions and experiences regarding hypertension management qualitatively. The TDF served as a conceptual framework to inform the design of the study. The use of TDF enhanced the robustness, methodological rigor, and applicative relevance of findings [65]. On the other hand, this study had some limitations. Firstly, the interviews were conducted in English only; however, none of the physicians interviewed reported any linguistic difficulties in reading or understanding English, and English is considered one of the core languages for communication in Qatar. In addition, not all participants who were contacted responded, and some responded but refused to participate, subsequently there is a possibility of nonresponse bias. Yet, based on the demographics of respondents, it is clear that they are representative of the population of primary care physicians in Qatar, confirming the external validity of the study.

## Conclusions

In conclusion, the interviews revealed that physicians’ knowledge, skills, goals, and beliefs about capabilities and consequences based on patient, medication, and organization factors, along with behavioral regulation, resources, and perceived sociopolitical and organizational context were the main factors that affect their prescribing of antihypertensives. Among their perceived barriers for hypertension management, they considered lack of consultation time and insufficient availability of certain antihypertensives in primary care. On the other hand, several facilitators were identified, and many strategies for improving hypertension management were suggested. These include the re-establishment of NCD clinics and offering education workshops for physicians and nurses on the management of hypertension. Further research is warranted within the primary care healthcare sector in Qatar to investigate the efficacy of NCD clinics on the management of chronic diseases. This should be accompanied by assessments of Continuous Professional Development (CPD) workshops focused on hypertension management and their impact on physicians’ competencies. Moreover, there is a need for additional investigation into the availability of antihypertensive medications in primary care centers across Qatar to determine potential formulary adjustments. Furthermore, research aimed at exploring the feasibility of extending consultation durations for physicians to enhance the management of chronic conditions is recommended.
